# The Behavior of *Rickettsia*-Positive *Dermacentor reticulatus* Ticks under Laboratory Conditions

**DOI:** 10.3390/life13030612

**Published:** 2023-02-22

**Authors:** Natália Pipová, Katarína Peňazziová, Miroslav Baňas, Igor Majláth, Viktória Majláthová

**Affiliations:** 1 Department of Animal Physiology, Institute of Biology and Ecology, Faculty of Science, Pavol Jozef Šafárik University, 041 80 Košice, Slovakia; 2 Department of Microbiology and Immunology, University of Veterinary Medicine and Pharmacy in Košice, 041 81 Košice, Slovakia

**Keywords:** *Dermacentor*, behavior, pathogens, parasitic manipulation

## Abstract

Considering that tick-borne diseases are on the rise, a full understanding of how pathogen–tick–host interactions can lead to vector behavioral modifications is of high value. Successful transmission of pathogens to their hosts depends on vector mobility and their ability to quest for prey and attack hosts. In our research, the behavior of *Dermacentor reticulatus* ticks (*n* = 88) that were positive or negative for *Rickettsia* spp. (prevalence—36.36%) were analyzed using novel behavioral apparatuses. Tick locomotion and their preference for light or darkness were studied utilizing a multirod arena (MA) and a light/dark test (LDT) optimized for ticks. Behavioral tasks were evaluated using a Smart video-tracking system (Panlab, Spain). The majority of ticks (*p* < 0.0001) explored in the center of MA during the first 15 min. Despite that, most of them (*p* < 0.01) chose the periphery for questing or resting postures. They also preferred the elevated parts of the rods (*p* < 0.001) for this behavior. Ticks significantly (*p* < 0.0001) preferred the light part of the LDT. *Rickettsia*-infected ticks had higher locomotor activity, manifesting as longer trajectories (*p* = 0.0221). Our results revealed the possible impact of pathogens on some behavioral patterns of ticks as their vectors, which may significantly increase the probability of pathogen transmission.

## 1. Introduction

Pathogens, causing many animal and human diseases, have developed a variety of adaptation and survival strategies within the host. Those that have achieved excellent host adaptations have a remarkably diverse spectrum of mechanisms to modify some phenotypic traits [1,2,3]. These mechanisms include, for example, modulating host immunity [4] and affecting host metabolism [5,6] in order to facilitate and prolong infection and maximize the probability of transmission. A notable category of phenotypic traits that can be dramatically changed is the behavioral alterations of vectors [7]. Some behavioral changes are only by-products of parasite presence, but other relatively significant ones can also occur as an outcome of host manipulation. Parasite-induced alterations in host behavior increase the chance for parasite survival by maximizing their fitness [1,2,3,7] and transmission or ensuring the completion of their life cycles [8].

Among the behavioral changes of a given arthropod, vectors infected with pathogens are characterized by higher biting rates [9,10,11,12,13], longer biting duration [14,15,16,17,18], increased host searching [19], an overall increase in their locomotor ability [20] boosting their chance to detect potential hosts, improvement in mating performance [21] or, conversely, reducing vector reproduction to increase nutrients available for the microorganisms and/or enhancing vector survival [22]. It has been emphasized that vector phenotyping trait manipulation is more common among parasites with a complex life cycle [19,23,24]. This may also be a strategy of pathogens responsible for tick-borne diseases. Our knowledge of how pathogens can alter the behavior of tick vectors is inferior to our knowledge of the behavioral changes widely investigated in pathogen–insect vector interactions [10,11,12,19].

Research on how pathogen infection can promote behavioral modifications in tick vectors has focused mostly on *Borrelia* spp., Lyme borreliosis’s agents. The locomotor activity was studied in both adult and immature *Ixodes* ticks infected with *Borrelia* spp. [25,26,27]. In the study by Alekseev et al. [25], locomotor activity of all stages of infected *I. ricinus* was suppressed when compared with non-infected specimens. In addition, the locomotor activity of *I. persulcatus* females with exoskeleton anomalies infected with *Borrelia* spp. was 1.3 times greater in comparison with infected ticks without anomalies [25]. It was also confirmed that *Borrelia*-infected *I. ricinus* nymphs had higher survival times under desiccating conditions and walked less within a humidity gradient [26]. In another study [27], the effects of *B. burgdorferi* infection in black-legged ticks (*I. scapularis*) were more obvious in adults than in nymphs. Infected adults were less able to overcome physical obstacles, avoided vertical surfaces, were less active, and quested at lower heights than uninfected ticks. Infected nymphs showed increased phototaxis, attraction to vertical surfaces, and (statistically not significant) trends of increased questing height and a greater tendency to overcome physical obstacles [27].

Only a few studies have been carried out on behavioral changes caused by *Babesia* spp., *Bartonella* spp., *Rickettsia* spp., and TBEV infection [7].

*Ixodes* ticks infected with *Anaplasma* spp. underwent significant physiological changes that influenced tick survival, questing, and feeding activity [28,29]. *Anaplasma phagocytophilum* infection modified the expression of key genes, which led to improvements in vector fitness and facilitation of pathogen transmission through increased glycolysis in the tick vector *I. scapularis* [28,30].

*Babesia microti* infection enhanced the feeding success and survival in tick *I. trianguliceps* [31]. Feeding time, engorged body weight, and molting rate influenced by *B. microti* infection in *I. scapularis* were also studied by Hu et al. [32].

*Bartonella henselae*-infected and non-infected female *I. ricinus* ticks were investigated to identify tick salivary gland factors, which are likely implicated in transmission [33]. In response to infection, the presence of the serine protease inhibitor (IrSPI), a member of the BPTI/Kunitz family of serine protease inhibitors, showed the highest upregulation. IrSPI silencing impaired tick feeding and thus reduced tick weight, in addition to initiating a reduction in bacterial load in tick salivary glands [33].

TBEV infection also showed a significant impact on tick behavior. TBEV-infected *I. ricinus* adults searching for a host were more active and tolerant than non-infected ones when exposed to growing concentrations of the N,N-diethyl-meta-toluamide (DEET) repellent. Furthermore, the titer of TBEV already present in unfed questing ticks increased to a detectible level after feeding [34]. TBEV infection also magnified tick mobility (walking speed and length of the trajectory) in *I. persulcatus* during host-seeking behavior [35]. In addition, *I. persulcatus* ticks infected with TBEV reached a higher questing height in comparison to uninfected ticks [36].

*Rickettsia* spp., which are small, obligate intracellular Gram-negative bacteria, cause illness throughout the world. They are transmitted by a variety of hematophagous arthropod vectors [37]. Most *Rickettsiae* are transmitted by ticks. The various species are endemic, and associated human clinical manifestations differ depending on the infectious agents and geographical locations [38,39]. Many new pathogenic *Rickettsiae* have been identified in recent years [38]. Despite its importance to public health, little is known about how the rickettsial infection may affect vector behavior. Fratczak et al. [40] studied the reaction of *I. ricinus* males and females in a 900 MHz electromagnetic field in a radiation-shielded tube. Ticks infected by *Rickettsia* spp., as well as those co-infected by *B. burgdorferi* s.l., were more attracted towards an irradiated area in comparison with the uninfected ones [40].

*Dermacentor reticulatus* ticks play an important role in the maintenance of pathogens causing human and animal diseases in the environment. They are the main vector of *Babesia canis*, the agent of canine babesiosis, one of the most threatening diseases for dogs in endemic regions [41]. The list of pathogens detected in *D. reticulatus* [42] includes pathogens where the vector role of these ticks is confirmed (Omsk haemorrhagic fever virus, tick-borne encephalitis virus, *Babesia canis, R. raoultii*, and *R. slovaca*, among others, with the latter two causing tick-borne lymphadenopathy—TIBOLA) or pathogens found in questing or fed ticks whose vector role is unknown (*Rickettsia helvetica*, *B. burgdorferi* s.l., *A. phagocytophilum*, *Coxiella burnetii*, among others). *D. reticulatus* is the second most important tick species (after *I. ricinus*) in many European countries, but is rare in the dry Mediterranean climate zone and is absent in cold Scandinavian countries [43]. Several studies indicated that the distribution of these ticks has expanded within Europe over the last several decades [41,44,45,46,47,48,49]. In comparison with *Ixodes* ticks, the behavior of *Dermacentor* ticks under the influence of pathogens has been insufficiently investigated.

Behavior in the field depends on many variables; therefore, a number of behavioral studies are carried out in laboratory conditions. Locomotor activity [50,51,52], questing behavior [53], influence of hydration on behavior [54,55], and repellent reactions [56,57] are among the behavior types that have been studied the most. The majority of research focuses on monitoring tick–stimulus response [51,54,57,58,59,60]. Apparatuses that are designed to test locomotor behavior and influence of hydration commonly have horizontal and vertical sections and wet areas [25,61,62]. Apparatuses for testing questing behavior and repellent reactions mostly have horizontal spheres and areas with repellent or attractants associated with hosts or live hosts [56,58,59,60,63].

The aim of our research was to describe the behavior of *D. reticulatus* ticks under laboratory conditions in devices designed in this study: a multirod arena (MA), enabling horizontal and vertical exploration to observe locomotor activity and position choice for questing or resting behavior, and a light/dark test (LDT) to find out the light/dark preference of the ticks. We investigated the behavioral differences of *D. reticulatus* ticks infected and uninfected with *Rickettsia* spp. The difference in the weight between *Rickettsia*-infected and *Rickettsia*-uninfected ticks was also examined.

## 2. Materials and Methods

### 2.1. Model Organisms and Experimental Design

*D. reticulatus* adult females (*n* = 88) were used in the experiments. Ticks were collected by flagging with a white cotton blanket (100 × 80 cm) in two areas in Slovakia: Ladmovce (*n* = 30; an altitude of 100 m.a.s.l.; 48° 24′14.46″ S; 21° 46′8.82″ W) and Jablonov nad Turňou (*n* = 58; an altitude of 198 m.a.s.l.; 48° 35′22.56″ S; 20° 41′47.44″ W). They were maintained under standard conditions (temperature—16 °C, humidity—90%). Behaviors of ticks were assessed using behavioral methods under laboratory conditions. They were tested in the MA for 24 h and in the LDT for the next 24 h. After completing the behavioral testing, they were weighed using analytical scales and then subjected to molecular analyses for selected pathogens.

### 2.2. Multirod Arena

**Figure 1:**
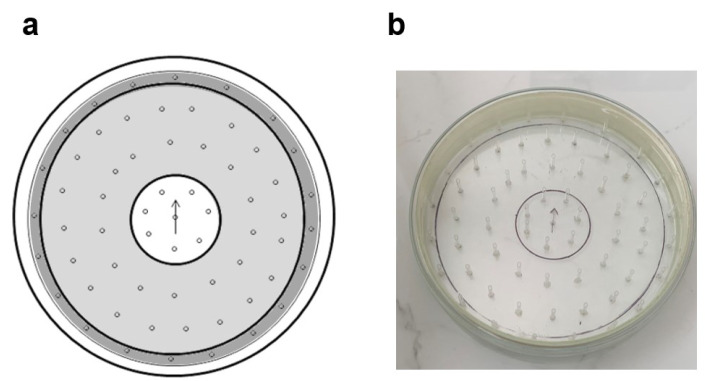
Multirod arena (MA). (**a**) ◦—skewed rods (glass capillaries); white—center with arrow determining the orientation of the slope of the skewed rods; light gray—internal; dark gray—border. (**b**) Photo of the MA.

Inspired by the open field test, which was developed by Calvin S. Hall in 1934 [64] and used to assay general locomotor activity levels, anxiety, and willingness to explore in rodents, we designed the MA (Figure 1) to investigate locomotor activity in ticks. The MA used in the bioassays consisted of an 18.5 cm-diameter glass Petri dish and 62 (1.5 cm-long) glass capillaries to allow vertical movement. Two concentric circles, 5.5 cm and 17 cm in diameter, were drawn on the outer surface of the Petri dish to divide the center (C), internal (INT), and border (B) areas. Capillaries were placed into 4 circles. The capillaries were skewed using the water sandpaper. They were fixed with aquarium silicone, with the bevel in the same direction. Capillaries did not exceed the height of the Petri dish and thus ensured comfortable questing/resting of the ticks. The side wall of the Petri dish was covered with a white tape to indicate the end of the field for exploration. The Petri dish was covered with a glass lid to prevent ticks escaping. Before each test, the apparatus was wiped with 70% ethanol to abolish odor cues, which was left to evaporate. Ticks were placed individually in area C and were tested individually for 24 h.

### 2.3. Light/Dark Test

**Figure 2:**
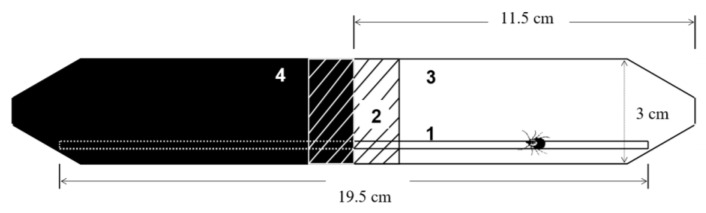
Light/dark test: 1—wooden stick; 2—rubber ring; 3—transparent tube; 4—black tube.

The LDT that allowed us to test tick preference for light or dark (Figure 2) consisted of two 50 mL polypropylene tubes, one transparent and the other black-opaque. Tubes were connected by a rubber ring. A new wooden stick was placed horizontally in the arena for each subject to facilitate movement.

The ticks were inserted at the border of the light and dark part of the LDT and left for 24 h. In order to abolish odor cues, the apparatus was wiped with 70% ethanol before each test. After finishing the test, the ticks were placed in Eppendorf micro tubes filled with 80% ethanol.

### 2.4. Behavioral Assay and Ambient Conditions

The test room was constantly illuminated during experiments. In order to decrease light intensity and to ensure the constant light conditions, window shades and ceiling lights were installed. The effect of the shadow that is preferred by ticks was achieved [65]. Shades also eliminated the unwanted reflection of light from the glass device to the camera. Since both apparatuses were closed, external stimuli were also eliminated, including different humidity levels or potential host attractants. The temperature during testing was recorded using data loggers located directly in the apparatus. The temperature ranged from 18 to 21 °C.

**Figure 3:**
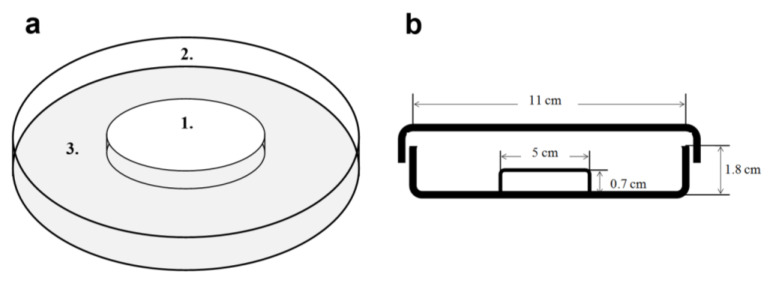
Hydration arena (**a**): 4.5 cm-diameter glass Petri dish (1) placed upside-down in a larger (11 cm-diameter) Petri dish (2) containing a wet filter paper (3) and covered with the glass lid. (**b**): Side view.

Before testing in the MA, ticks were allowed to hydrate at room temperature in the hydration arena (HA, Figure 3) for 24 h. Consequently, a comparable level of hydration in ticks was achieved. The behavior in the MA was video-recorded and evaluated using the Smart 3.0 computerized video-tracking system (Panlab, Spain) over the first 15 min, during which the habituation phase took place. Parameters of locomotor activity determining the level of exploratory behavior—distance travelled and the latency of entry from C to other parts of the apparatus (INT and B)—were analyzed.

After 24 h, most of the ticks were in a resting posture or questing attitude, which was recorded as their final position. If they were still in active locomotion, they were left longer. After completion of the MA test, ticks were transferred into the LDT for further testing.

Behavioral testing procedure:

1. Hydration of ticks in HA for 24 h.
2. Testing the ticks in the MA.
3. Recording tick movement with the camera for the first 15 min.
4. Recording the final position of the tick in the MA after 24 h (or longer).
5. Testing the ticks in the LDT.

### 2.5. Body Weight

Ticks were removed from ethanol, dried on filter paper, and weighed using analytical scales.

### 2.6. Molecular Detection of Pathogens

The ticks (*n* = 88; Ladmovce *n* = 30, Jablonov nad Turňou *n* = 58) were examined using molecular analysis to determine the presence of pathogens of the genus *Rickettsia* spp., *Ehrlichia* spp., and *Babesia* spp. DNA extraction was accomplished using a phenol-chloroform method. EHR 521 and EHR 747 primers were used to amplify part of 16S rRNA gene of the family Anaplasmataceae [66]; BN1 and BJ2 primers were used to target fragments of 18S rRNA for the detection of *Babesia* spp. [67]; and D 767 and D 1390 primers were used to amplify surface cell antigen 4 for the detection of *Rickettsia* spp. [68].

### 2.7. Statistical Analysis

To see whether the proportions of final position (top vs. bottom) and parts of apparatuses (C vs. INT vs. B; light vs. dark) differ in ticks and between infected and uninfected ticks, we used Pearson’s chi-squared test. Non-normal distributed continuous data (time spent in particular parts of the arena, latency to enter INT and B, distance travelled) were analyzed using the Kruskal–Wallis test, Dunn’s multiple comparison post hoc test, and the Mann–Whitney test. Differences were considered significant at *p* < 0.05. The statistical analyses were performed using the Prism 7 (Graph Pad Software Inc., San Diego, CA, USA).

## 3. Results

### 3.1. Ticks in the Multirod Arena

In total, the records of behavior of 72 *D. reticulatus* female ticks were suitable for evaluation.

70.83% of ticks (51 of 72, *p* < 0.0001) were in active locomotion during the recording time. We referred to them as “active ticks”.

**Figure 4:**
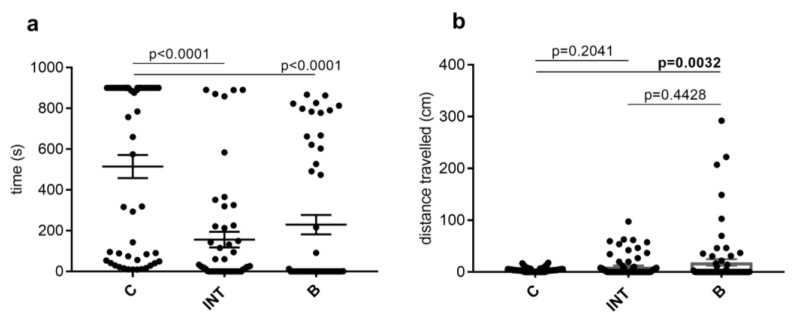
(**a**): Time which *Dermacentor reticulatus* ticks spent in individual parts (C—center, INT—internal, B—border) of the multirod arena (MA). (**b**): Trajectories travelled by ticks in C, INT, B of the MA.

During recording time (the first 15 min), the ticks spent most of the time in C compared to INT (*p* < 0.0001) and B (*p* < 0.0001); H_3216_ = 99.15, *p* < 0.0001. We obtained similar results when we evaluated the time spent in individual parts of the apparatus by active ticks only; H_3153_ = 43.02, *p* < 0.0001 (Figure 4a).

The Kruskal–Wallis test also revealed differences in trajectories travelled in these parts (H_3216_ = 10.76, *p* = 0.0046). Dunn’s multiple comparison post hoc test showed significantly longer trajectories travelled in B only (*p* = 0.0032) in comparison with trajectories in C (Figure 4b).

The ticks chose a final position mainly at the top of the rods, which indicated a significant preference (*p* = 0.0004) for elevated places for questing attitude or resting position.

Within the individual parts of the apparatus, the ticks chose B significantly more (*p* < 0.01) than INT for the final position.

### 3.2. Rickettsia-Infected vs. Rickettsia-Uninfected Ticks

The presence of *Rickettsia* spp. and *Ehrlichia* spp. pathogens was confirmed in *D. reticulatus* ticks. None of the samples were positive for *Babesia* spp. DNA of *Rickettsia* spp. was identified in 36.36% (32 of 88; Ladmovce *n* = 5, Jablonov nad Turňou *n* = 27). The prevalence of *Ehrlichia* spp. was 5.68% (5 of 88; Ladmovce *n* = 3, Jablonov nad Turňou *n* = 2). No cases of co-infection were detected. The impact of pathogens on the behavior was evaluated only in ticks infected with *Rickettsia* spp. due to the low number of *Ehrlichia*-infected ticks.

The number of active ticks (ticks that were in active locomotion during the recording time) across infected and uninfected ticks was non-significant: χ2 = 0.121, 1 df, *p* = 0.7279.

**Figure 5:**
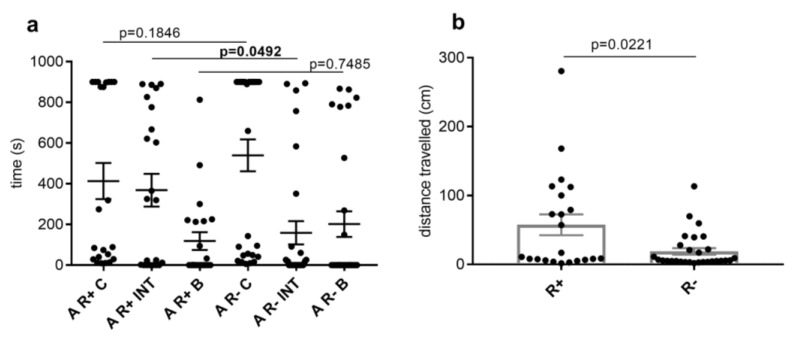
(**a**): Time which *Dermacentor reticulatus* ticks, infected (R+) and uninfected (R−) with *Rickettsia* spp., spent in individual parts (C—center, INT—internal, B—border) of the multirod arena (MA). These data include only ticks that were active in locomotion during recording time (the first 15 min). (**b**): Trajectories travelled by R+ and R− ticks in C, INT, B of the MA.

The time spent in the individual parts of the MA by ticks did not differ significantly. However, when comparing the time spent in the parts of the apparatus only in *Rickettsia*-infected and uninfected ticks, which were active in locomotion, a significant difference was observed. Infected ticks spent significantly more time than uninfected ticks (*p* = 0.0492) in INT (Figure 5a).

We also recorded significantly longer (*p* = 0.0221) distances travelled during the first 15 min in *Rickettsia*-infected ticks (Figure 5b).

The latency of the entrance to INT and B was also compared as a parameter of locomotor activity in *Rickettsia*-infected and uninfected ticks. The maximum value for statistical analysis was set at 900 s (recording time). No significant difference was revealed (latency to INT, *p* = 0.2469; latency to B, *p* = 0.3737).

The chi-squared test showed that there were no significant differences in final position preference between ticks infected and uninfected with *Rickettsia* spp.:

top vs. bottom: χ2 = 0.3064, 1 df, *p* = 0.5799
C vs. INT vs. B: χ2 = 0.9321, 2 df, *p* = 0.6275.

### 3.3. Ticks in the Light/Dark Test

**Figure 6:**
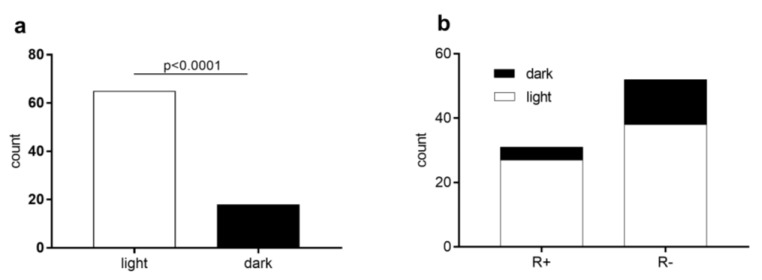
(**a**): The light/dark preference of *Dermacentor reticulatus* ticks in light/dark test (LDT). (**b**): Light/dark preference in *Rickettsia*-infected (R+) and uninfected (R−) ticks in LDT.

The majority of ticks (65 of 88) preferred the light part of the apparatus: χ2 = 2171, 2 df, *p* < 0.0001 (Figure 6a). *Rickettsia*-positive and *Rickettsia*-negative ticks evaluated separately also had significant preference for the light part (*p* < 0.0001). Since five ticks were found on the border, they were excluded from the statistic. We did not record any significant difference in the light/dark preference in *Rickettsia*-positive vs. *Rickettsia*-negative ticks: χ2 = 2.248, 1 df, *p* = 0.1338 (Figure 6b).

### 3.4. Body Weight

The weight of the ticks ranged between 30–40 × 10^−4^ g. The average weight of ticks from Ladmovce was 40 × 10^−4^ g, and from Jablonov nad Turňou it was 48 × 10^−4^ g. No significant difference between the weight of *Rickettsia*-infected and uninfected ticks was demonstrated. The average weight of the infected ticks was 46 × 10^−4^ g; the average weight of the uninfected ticks was 45 × 10^−4^ g.

## 4. Discussion

Ticks are very important vectors for many pathogens which cause various animal and human diseases. The aim of our research was to study the behavioral activity of *D. reticulatus* ticks under controlled laboratory conditions. We designed two apparatuses for this purpose. The MA was created to investigate locomotor activity, exploratory, questing, and resting behavior. The LDT was used to test the light/dark preference of the ticks.

The first 15 min of tick behavioral response in their new MA environment were important for analyzing the latency time that ticks needed to enter the MA zones, the time spent in the individual parts of the apparatus, and the travelled distance. This habituation phase was continuously followed by the phase in which we left ticks in the arena for up to 24 h. During this phase, the ticks were choosing places for questing or resting.

The questing/resting position was recorded mostly on the top of the glass capillaries. This indicates a considerable preference for elevated positions. We assume that this preference is advantageous for the ticks in allowing them to more quickly and efficiently grip on the potential host while also protecting them from predators. In the experiments of Dawes-Gromadzki and Bull [69], ticks in leaf litter were more protected from predation by ants than those on bare soil.

Compared to the other zones of the arena, ticks spent most of the recording time in C. Despite the fact that only ticks active in locomotion were inserted into the MA, some ticks (29.17%) did not move after insertion into the apparatus, so they spent the whole time in C. We therefore also analyzed only the active ticks with the tendency to explore (ticks that were at least in a minimum active locomotion during recording time). The analysis showed that active ticks also spent most of the time in C during the first 15 min.

Based on the results obtained from the analysis of the time spent in the particular parts of the apparatus, we expected that ticks would choose, for the final position, the most exposed part of the arena (C). The assumption has not been confirmed, since the highest number of final positions and the longest trajectories were recorded in B. This finding showed that ticks were exploring the arena in subsequent hours.

Due to the low number of *Ehrlichia*-infected ticks, the impact of pathogens on the behavior was evaluated only in ticks infected with *Rickettsia* spp. The behavior modifications of pathogen-infected ticks have been the subject of several investigations [25,55,70]. Alekseev et al. [25] and Herrmann and Gern [55] focused mainly on the influence of *B. burgdorferi* s.l. on the behavior of *I. ricinus* ticks. The results of both studies indicated that ticks infected with these bacteria were less active in locomotion and responded better to dry environments [25,55]. Cruz et al. [70] studied how pathogens increase the fitness of ticks as their vectors. They point out that the infected ticks better handled environmental demands. *I. scapularis* ticks infected with *A. phagocytophilum* had significantly increased resistance to frost and HSP production [70].

In our research, we observed a significant increase in some activity parameters of the tested ticks, which were most likely caused by *Rickettsia* spp. Increased exploration activity of *Rickettsia*-positive ticks was reflected by longer trajectory compared to uninfected ticks. Similarly, dengue virus-infected *Aedes aegypti* mosquitoes showed an overall increase in their locomotor ability [20]. Higher locomotor activity can boost the vector’s likelihood to detect potential hosts and to spread pathogens more effectively. Possible impacts of *Rickettsia* spp. infection on the tick’s activity were shown in the opposite way, as in the case of *B. burgdorferi* s.l. infection, which caused a decrease in locomotion activity [25,55]. This difference could be due to the different requirements that bacteria have for their spread.

The behavioral differences between infected and uninfected ticks that were active in locomotion were also observed when comparing time spent in individual zones. *Rickettsia*-positive ticks spent more time in INT than *Rickettsia*-negative ticks.

Since *D. reticulatus* ticks possess developed visual organs, we were interested to see whether they preferred illuminated or shaded places. It is interesting to note that ticks that have been exposed to three-day lighting mostly preferred light parts of the LDT. The modification of behavior between *Rickettsia*-positive and negative ticks was not confirmed in LDT. Our results correspond with the findings of Lees [62], whose research on the light sensitivity of ticks indicates that ticks that were exposed to light gradually did not avoid it. He also claims that the older ticks avoided the light less [62]. According to the study by Bartosik et al. [71], the spring and autumn daily activity of *D. reticulatus* ticks peaked around 2 p.m., then decreased as twilight approached. This decline in activity may be explained by the host itself, whose biological properties (e.g., circadian activity) greatly influence the behavioral manifestations of ticks [71]. Based on these findings, we can assume that ticks chose a light part of LDT because of greater chances of meeting a potential host. These conclusions are also supported by the study of Godfrey et al. [65], who observed the night activity of *Amblyomma sphenodonti* ticks, in contrast to *D. reticulatus* ticks. The difference between these species arises in their typical hosts. *A. sphenodonti* parasites on lizard-like reptiles from Sphenodontia order that are mainly active at night. *D. reticulatus* parasites on forest ruminants and dogs, which are particularly active during the light part of the day [65].

The influence of tick-borne pathogens on the energy reserves of ticks has also been studied. Ticks use their energy reserves to maintain water balance, promote host-seeking behavior, and spread infection. Several studies [26,72] confirmed that parasites of arthropods can affect the resource levels of their hosts. *B. burgdorferi* s.l.-infected *I. ricinus* nymphs had higher fat content when compared to uninfected ones; however, no relationship was revealed between spirochete load and fat content [26]. Similarly, Gassner [72] also observed that *B. afzelii*-infected *I. ricinus* nymphs had higher fat content than uninfected ones. This increase in fat content in *Borrelia*-infected nymphs is explained as a result of tick–host interactions (blood feeding), tick physiology (digestion and molting), and/or tick behavior that conserves fat reserves [26]. Energy generating from these reserves may be used for questing behavior and/or maintenance of water balance [26]. This is consistent with decreased locomotor activity recorded in *Borrelia*-infected ticks [25,27]. In contrast, we did not reveal a significant difference in weight between *Rickettsia*-infected and uninfected ticks in our experiment. The behavioral response of ticks infected with *Rickettsia* spp. was also contrasting, which may indicate a different strategy of this type of pathogen for transmission and spreading in the environment.

Understanding the behavioral manifestations of ticks can contribute to the development of effective procedures of infection prevention. In our work, we focused on the research of these manifestations in laboratory conditions. We analyzed the behavior of ticks and the behavior of pathogen-infected ticks. Our results revealed the possible influence of pathogens on some behavioral patterns of ticks as their vectors. We developed the apparatuses, which offer additional possibilities for investigating the various behavioral responses in species other than ticks.

In our experiment, we monitored behavioral activities that were not influenced by external odor stimuli. However, thanks to the presence of a large number of hollow glass capillaries, the MA can be used to monitor the influence of odors on the behavior of arthropods. Various attractants or repellents inserted into these capillaries could specifically affect the behavior of studied organisms. Behavioral research on vectors still offers a wide range of options. Sufficient understanding of factors determining transmission, which undoubtedly includes the behavior of vectors, can improve infectious disease control and prevention.

## Data Availability

Not applicable.
